# Engineered barriers regulate osteoblast cell migration in vertical direction

**DOI:** 10.1038/s41598-022-08262-5

**Published:** 2022-03-15

**Authors:** X. Chen, Y. Xu, Y. Cheng, S. W. Pang

**Affiliations:** 1 Department of Electrical Engineering, City University of Hong Kong, Kowloon, Hong Kong China; 2 Centre for Biosystems, Neuroscience, and Nanotechnology, City University of Hong Kong, Kowloon, Hong Kong China

**Keywords:** Biomedical engineering, Biotechnology

## Abstract

Considering cell migration is essential for understanding physiological processes and diseases. The vertical migration of cells in three dimensions is vital, but most previous studies on cell migration have only focused on two-dimensional horizontal migration. In this paper, cell migration in the vertical direction was studied. Barriers with a height of 1, 5, 10, and 25 µm with grating and arrows in channels as guiding patterns were fabricated. The effects of barrier height and guiding patterns on the vertical migration of MC3T3 cells were explored. The study revealed that taller barriers hinder vertical migration of MC3T3 cells, whereas grating and arrows in channels promote it. The time-lapse and micrograph images showed that as the barrier height increased, the cell climbing angle along the barrier sidewall decreased, and the time taken to climb over the barrier increased. These results indicate that taller barriers increase the difficulty of vertical migration by MC3T3 cells. To promote the vertical migration of MC3T3 cells, 10 µm tall barriers with 18° and 40° sloped sidewalls were fabricated. For barriers with 18° sloped sidewalls, the probability for MC3T3 cells to climb up and down the 10 µm tall barriers was 40.6% and 20.3%, respectively; this is much higher than the migration probability over vertical barriers. This study shows topographic guidance on the vertical migration of MC3T3 cells and broadens the understanding of cell migration in the vertical direction.

## Introduction

Cell migration occurs in physiological processes, such as embryonic development, wound healing, and immune responses, and is a fundamental part of life^1–4^. It is also essential in cancer metastasis, which results in cancer morbidity and mortality^5,6^. Thus, the study of cell migration is essential for understanding diseases^7^. In addition to subcellular mechanisms, which include microtubule disruption and regeneration, signaling processes, and genetic analysis^8–11^, interactions in the extracellular matrix (ECM) play a significant role in regulating cell migration^11,12^. The ECM regulates cell migration through chemical, mechanical, and morphological cues^12–16^. Surface morphology, which can be engineered by advanced fabrication techniques, has proved to be an effective way to modulate cell migration^17–19^. For instance, unidirectional migration of MC3T3 cells has been reported by using engineered “V-shaped grating patterns”^20^. On platforms with varying pattern densities, cells prefer to migrate from areas with lower pattern density to higher pattern density^21^. On dense ridges, cells elongate and migrate along the ridges^22^.

However, most studies focused on investigating horizontal migration, and few reports have mentioned cell migration in the vertical direction, including those employing a three-dimensional (3D) matrix^23–25^. In most cases, researchers cultured cells in 3D matrices, which are produced with collagen, nanofibers, fibronectin, or polyethylene glycol-based hydrogels^26^. The interaction between cells and the 3D matrix was studied, specifically mechanics and signaling dynamics that cue cell migration^11,27,28^. Differences between cell migration on 2D platforms and 3D matrices due to changes in morphology were reported. Cells in a 3D ECM assume stellate morphology. On a 2D surface, they spread freely on the horizontal plane but not in the vertical direction^26^. Vertical migration has been reported by culturing cells in collagen gel and observing the vertical movement of cells by histochemical staining^7,29,30^. However, few studies separate cell migration in horizontal and vertical directions^31–33^. A confocal microscope is usually utilized to observe cells in a 3D matrix with 3D images^34^. However, results on dynamic tracking of cell migration vertically in a 3D matrix are minimal. Moreover, microenvironment influences on 3D culture are studied by controlling topographic cues on the horizontal plane^26^. Gradient concentration of dexamethasone and poly-d-lysine-functionalized periodic mesoporous organosilicas was reported to guide fibroblast cell migrate from bottom to the top of scaffold^33^, there is little information available for topographic cues to guide vertical cell migration in a 3D matrix, as vertical guidance is difficult to construct.

Cells moving in tissues typically experience complex ECM structures that force them to constantly switch between horizontal and vertical migration modes^27^, Physiological processes such as wound healing, cerebellum development, and cancer metastatic all involve both horizontal and vertical cell migration. For instance, vertical migration of fibroblast cells from bottom to top (dermis to the epidermis) is crucial during wond healing process. Malignant melanocytes (melanomas) metastatic processes start with vertical cell migration through underlying dermis on the skin. 3D migration of osteoblasts towards injury site from bone surface or mesenchymal envelope is important for bone remodeling, as the subtle coordination between osteoclasts’ and osteoblasts’ vertical and horizontal migration during bone remodeling ensures bone shape and structure remain stable^35^. Vertical migration of osteoblasts can also be an essential process of osteocytic differentiation to form the bone matrix^36^. Therefore, understanding the vertical transition phase of osteoblasts migration is essential, which could lead to valuable knowledge in bone remodeling. In this study, barriers with various heights were fabricated, and guiding patterns were formed to promote vertical migration. Grating and arrows in channels were utilized to guide cell migration towards the barriers. Live cells were tracked dynamically to follow cell migration vertically using time-lapse imaging. It was found that the grating helped vertical migration when the barriers were less than 10 µm tall, while arrows in channels promoted vertical migration over taller barriers. Furthermore, more cells climbed up than climbed down. In order to further promote vertical migration, the effect of engineered barriers with sloped sidewalls was studied. Small slope angles facilitated cell migration vertically. This study explores the vertical migration of cells on engineered platforms with guiding patterns, broadening the understanding of topographic guidance of cell migration in the vertical direction.

## Materials and methods

### Fabrication of engineered platforms with barriers and guiding patterns

**Figure 1:**
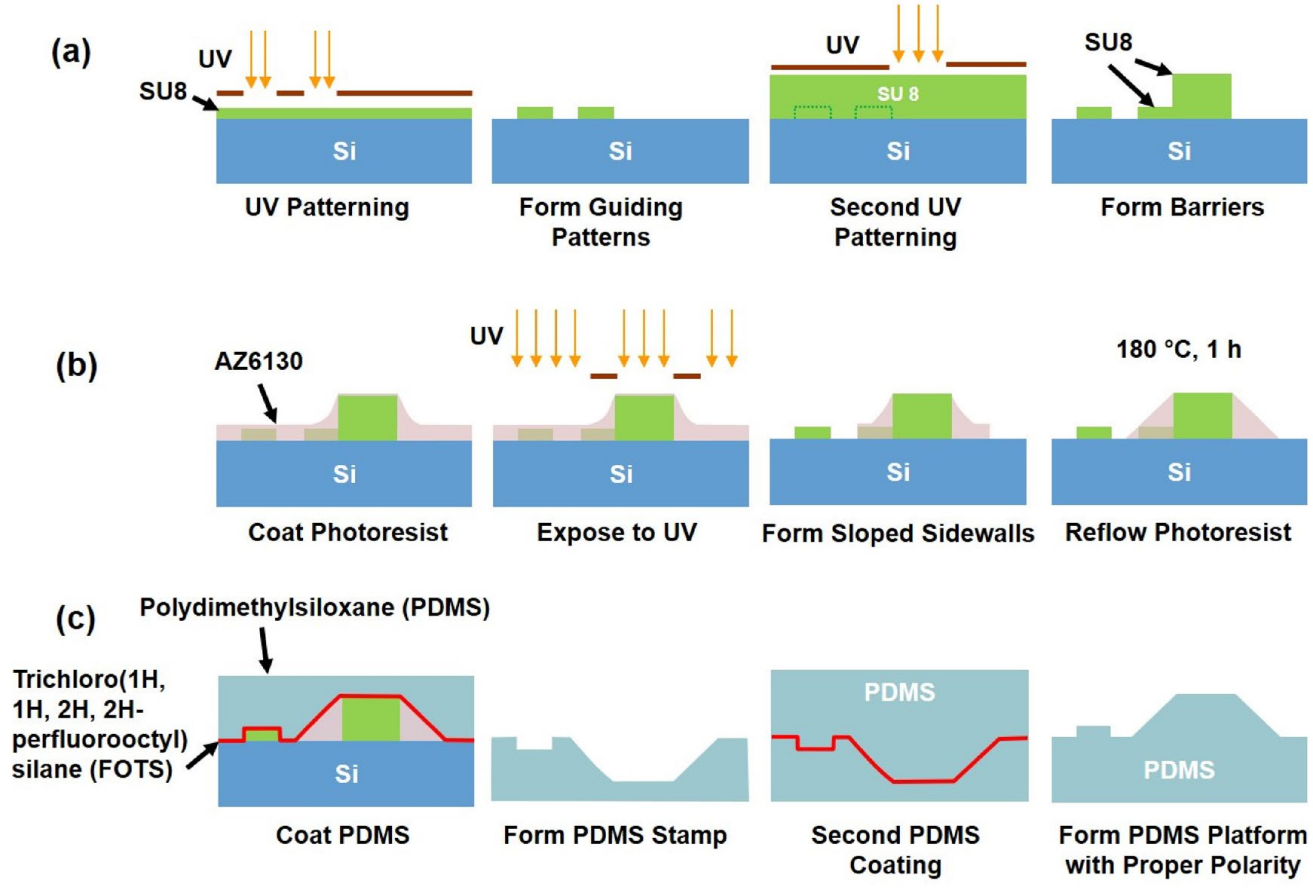
Schematic of fabrication technology of PDMS platform with barriers and guiding patterns. (**a**) Photolithography to fabricate grating and arrows in channels with different barrier heights. (**b**) Barriers with sloped sidewalls fabricated by photolithography and photoresist reflow. (**c**) Replication of PDMS platform from stamp with barriers and patterns of grating and arrows in channels.

Fabrication technology of the engineered platforms with barriers and guiding patterns is summarized in Fig. 1. Photolithography with ultra-violet (UV) light was employed to fabricate guiding patterns together with the engineered barriers. As shown in Fig. 1a, 1 µm thick SU8 photoresist was spin-coated on a Si wafer to form 5 µm wide grating with 5 µm spacing as a guiding pattern. In addition, arrows of 45° angle, 10 µm width, and 26 µm length with 26 µm spacing between arrowheads, 20 µm wide channel walls, and 10 µm spacing between channel walls with arrows in between were formed in SU8 as guiding pattern. Patterns in SU8 were fabricated by baking at 65 °C for 2 min and 95 °C for 3 min, exposure to UV light for 20 s, and development for 30 s. According to previous works^20,37,38^, 1 µm thick, 5 µm wide, and 5 µm spacing straight grating as well as pattern of arrowheads with 45° angle show good guidance on MC3T3 cell migration. In addition, previous study^38^ showed that cells had more lamellipodium extensions on grating patterns with acute angles. There were more constrains on lamellipodium formation when the acute angle of the triangular patterns was increased from 60° to 90°. In order to provide better cell migration guidance, 45° angle is chosen in this paper.

A second coating of another SU8 layer on the patterned wafer was performed following hard baking at 150 °C for 10 min and an O_2_ plasma treatment for 3 min with 20 mTorr chamber pressure, 20 sccm O_2_ flow rate, and 100 W radio frequency power. By adjusting the spin-coating speed and viscosity of SU8, 1 to 25 µm thick SU8 layers were obtained to form barriers with different heights. For the arrows in channels pattern, when the barriers were 1, 5, or 10 µm tall, 12 µm tall and 20 µm wide channels walls were fabricated after the barriers were formed by one more photolithography step. When the barrier was 25 µm tall, the 12 µm tall and 20 µm wide channel walls with arrows were generated before the 25 µm tall barrier was formed.

Next, a 5 µm thick AZ 6130 photoresist was spin-coated on the 10 µm tall SU8 barrier with grating, as shown in Fig. 1b. 10 and 30 µm wide AZ 6130 strips were produced beside the SU8 barriers through photolithography. The longer strips next to the barriers allowed longer extensions of shallower sloped sidewalls to be formed. After baking at 180 °C for 1 h, the 10 µm wide photoresist strips reflowed and formed 12 µm long, 40° sloped sidewalls and the 30 µm wide photoresist strips formed 35 µm long, 18° sloped sidewalls next to the barriers^39^.

Figure 1c shows the double casting of polydimethylsiloxane (PDMS) to replicate the patterns and barriers from the stamps fabricated in Figs. 1a,b, A layer of trichloro(1H, 1H, 2H, 2H-perfluorooctyl)silane (FOTS) was coated on the stamps after O_2_ plasma treatments. PDMS was then poured on the stamp and baked at 80 °C for 5 h to obtain the first PDMS stamp with the opposite polarity. A second O_2_ plasma treatment and FOTS coating were performed on the replicated PDMS stamps. A second PDMS coating was cast on the replicated PDMS stamp and baked at 80 °C for 5 h. Final PDMS platforms of the designed patterns with grating and arrows in channels, as well as engineered barriers with various heights and sloped angles, were obtained with the desired polarity. The PDMS platforms were then treated with an O_2_ plasma with 400 sccm O_2_, 400 sccm N_2_, 0.2 mbar pressure, and 200 W RF power for 1.5 min to form a hydrophilic surface, which is preferable for cell adhesion and proliferation.

### Cell culture and time-lapse imaging

MC3T3-E1 mouse osteoblast cells were cultured in Dulbecco’s modified Eagle medium (DMEM, high glucose, Gibco) at 37 °C in the humidified incubator with 5% CO_2_. The DMEM medium was supplemented with 10% fetal bovine serum (FBS, Gibco, Brazil), 1% alanyl-L-glutamine (GlutaMAX, Gibco), and 1% antibiotic–antimycotic (Penicillin–Streptomycin-Glutamine, Gibco). The cells were passaged before the confluency reached 90%. The DMEM medium was refreshed every 2–3 days.

The patterned PDMS platforms were bonded onto an O_2_ plasma treated, 35 mm diameter confocal dish, followed by heating at 80 °C for 3 min. The PDMS platforms together with the confocal dish were treated with an O_2_ plasma and kept in distilled water to maintain the hydrophilicity. After washing the PDMS platforms on the attached confocal dish with 70% ethanol and 0.01 mol/l phosphate buffered saline (PBS) twice, MC3T3-E1 cells with a density of 2.8 × 10^4^ cells/ml were added onto the platforms. Time-lapse imaging was conducted over a 16 h period after 6 h incubation using a Nikon upright microscope. In order to keep the cells healthy during imaging, they were cultured in a CO_2_-independent medium (Gibco) with 10% FBS, 1% GlutaMAX, and 1% Penicillin–Streptomycin-Glutamine supplement inside a 37 °C humidified incubator. Time-lapse images were captured every 5 min.

### Immunofluorescence staining and confocal microscopy

MC3T3-E1 cells were fixed after 24 h of incubation on the platforms. Cells were permealized by 0.1% Triton X-100 (ThermoFisher) and blocked by 1% bovine serum albumin (ThermoFisher). Cells were then stained by a FAK100 actin cytoskeleton/focal adhesion staining kit (Merck) with standard operation protocol and stored in 0.01 mol/l PBS.

Confocal images were captured by a Leica Stellaris 8 microscope. Fluorescence signal of F-Actin, vinculin, and nucleus were imaged by 532, 488, and 405 nm laser, and marked by red, green, and blue color, respectively. Reflected bright field images were captured using 650 nm laser. A 20 µm range z-direction scan was performed to get the 3D images with a step resolution of 0.4 µm.

### Data analysis

ImageJ software (version 1.48) was used to analyze cell migration information obtained through time-lapse imaging. The number of cells climbing up and down the barriers was counted. Cells were only counted if their entire bodies had moved up onto the top of the barriers or went down to the bottom. Migration trajectories were tracked with a manual tracking plugin, from which migration speed was calculated. The climbing angle along the sidewall of the barrier was calculated by measuring the angle of the cell trajectory across the barrier sidewall as the cell migrated from the bottom to the top of the barrier. Climbing time was counted as the time from when the cell touched the bottom of the barrier to the time when the entire cell body was on top of the barrier. All data were obtained from at least 3 runs (N ≥ 20 for each run) and tested by the one-way ANOVA with Tukey’s honest significant difference post-hoc test.

### Scanning electron microscopy

MC3T3-E1 cells were fixed after 24 h of incubation on platforms. To dry them before imaging, the cells were treated with 0.01 mol/l PBS for 10 min and 0.0025 mol/l PBS for 5 min. After immersing the cells in deionized water for 10 min twice, ethanol with increasing concentrations (30%, 50%, 70%, 80%, 90%, 95%, and 100%) was used to replace the water inside cells gradually. A Leica critical point dryer was then used to dry the cells. PDMS platforms with dry cells were then coated with a thin layer of gold by thermal evaporation. A Hitachi SU5000 scanning electron microscope was used to capture high-resolution images of cells on various platforms.

## Results and discussion

### Engineered barriers with guiding patterns

**Figure 2:**
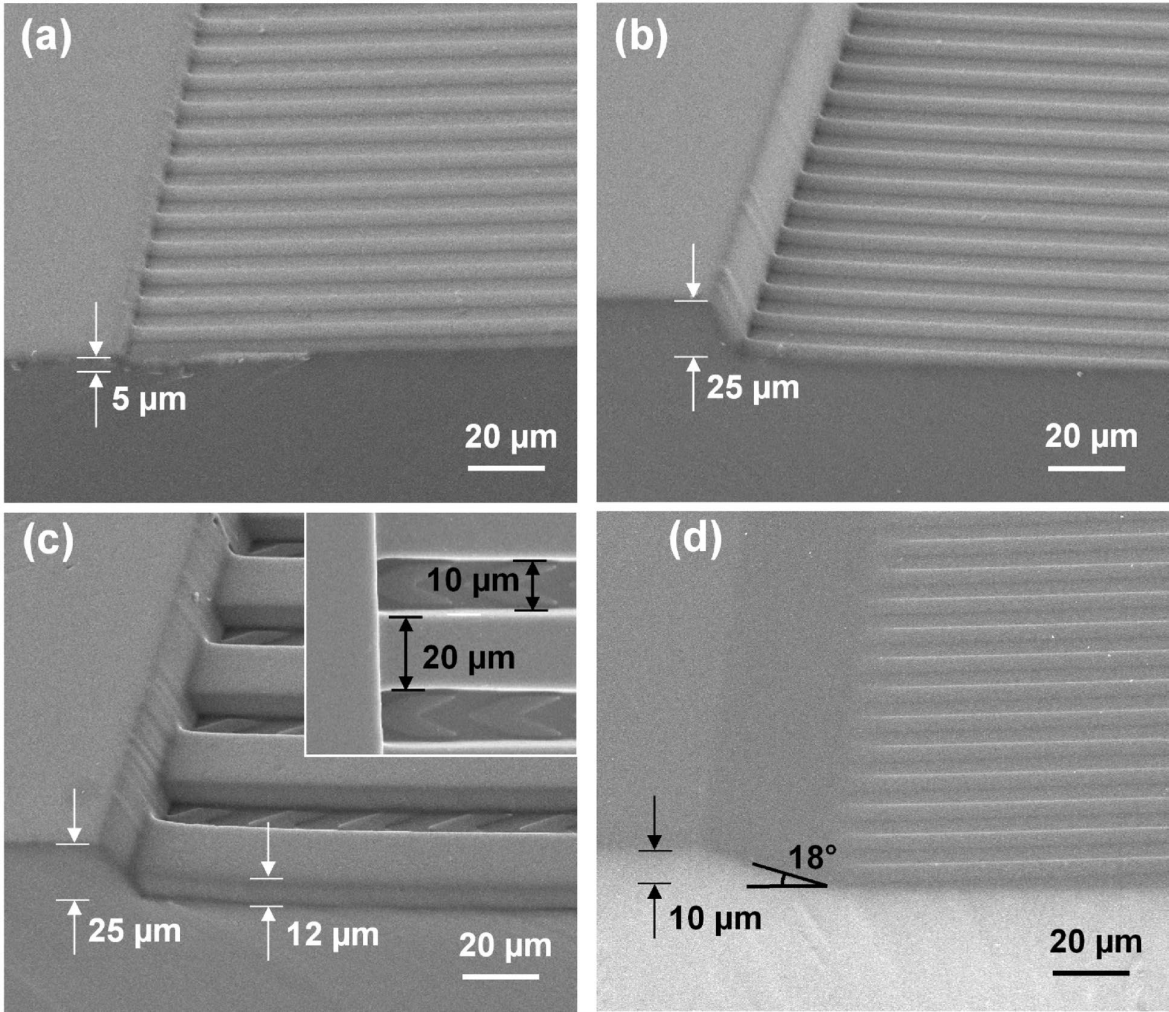
Scanning electron micrographs of PDMS platforms with 1 µm thick, 5 µm wide, and 5 µm spacing grating patterns with (**a**) 5 µm tall and (**b**) 25 µm tall barriers. (**c**) Arrows of 45° angle, 10 µm wide, 26 µm long, and 26 µm spacing between arrowheads in 10 µm wide channels with 20 µm wide and 12 µm thick channel walls next to 25 µm tall barrier. Inserted image on top right is top view of PDMS platform showing dimensions of grating width and spacing. (**d**) Grating with 10 µm tall, 18° sloped barrier.

In order to study the vertical migration of MC3T3 cells, PDMS platforms with increasing heights of 1, 5, 10, and 25 µm barriers were fabricated. Scanning electron micrographs of 5 and 25 µm tall barriers with a grating of 5 µm width, 5 µm spacing, and 1 µm thick are shown in Fig. 2a,b. As cells on flat surfaces migrate randomly and cells on grating migrate along the grating either towards or away from the barriers, it is necessary to form patterns to guide cells towards the barriers. Thus, arrows with confined channels were fabricated for guidance. The arrows pattern with a 45° angle, 10 µm width, and 26 µm length with 26 µm spacing between arrowheads in 12 µm tall, 20 µm wide channel walls, 10 µm wide channels between channel walls, and 25 µm tall barrier is shown in Fig. 2c. Moreover, to promote cell migration up the barriers, PDMS barriers with slope angles of 18° and 40° were fabricated. Figure 2d shows the 18° sloped sidewall and 10 µm tall barriers that extend 35 µm from the top of the barrier to the bottom next to a grating pattern.

### Effects of barrier height and guiding pattern on vertical cell migration

**Figure 3:**
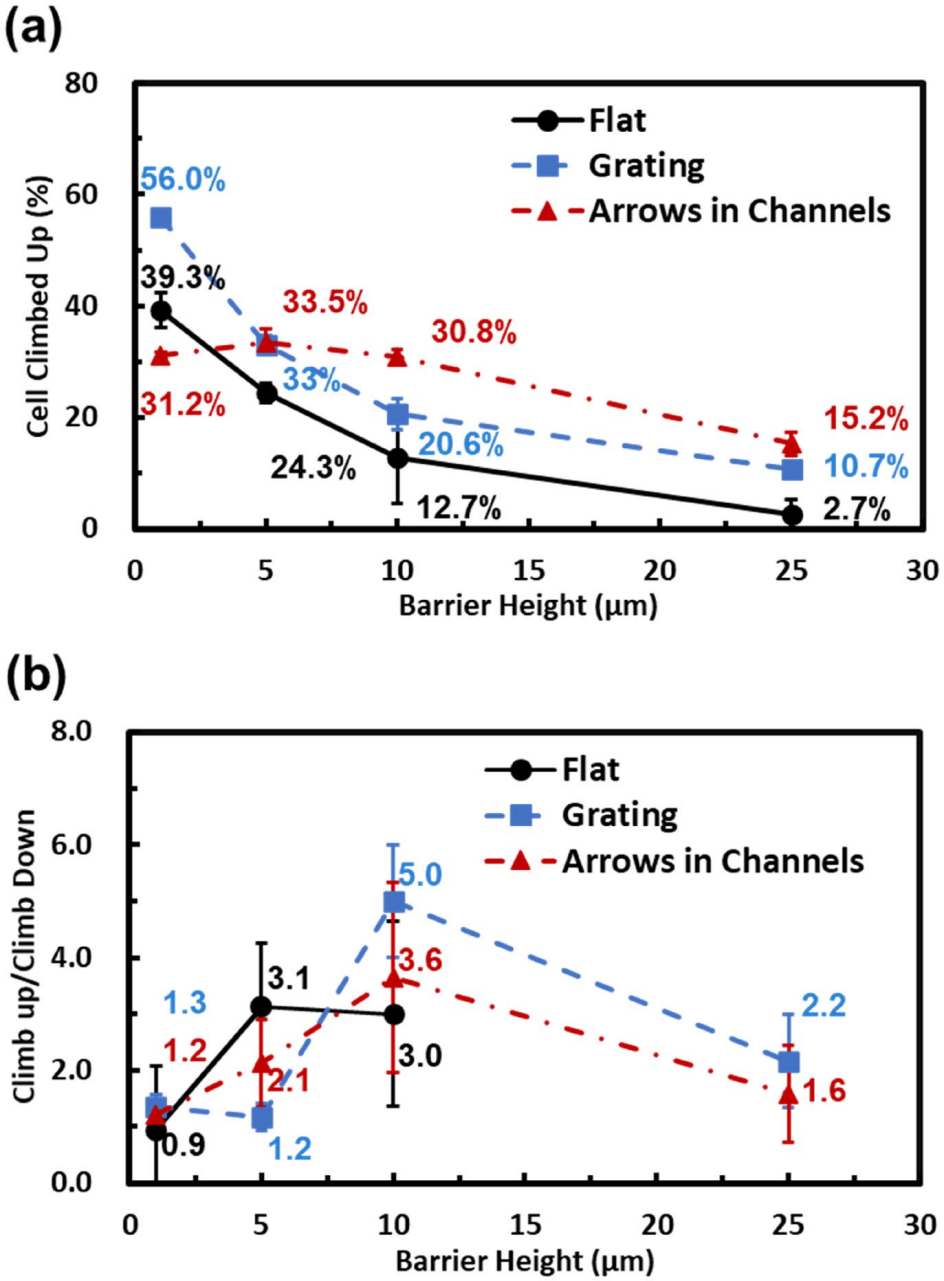
(**a**) Dependence of MC3T3 cells that climbed up on 1–25 µm tall barriers with grating and arrows in channels guiding patterns. (**b**) Ratio of MC3T3 cells climbed up vs. down of 1–25 µm tall barriers with various guiding patterns. All the data were obtained over a 16 h time-lapse imaging period.

When MC3T3 cells migrate and encounter a barrier, they will move in three different ways: climb vertically to the top of the barrier, move along the sidewalls of the barrier, or turn back. These three movements are shown in Supplementary Video SV1. The probability of MC3T3 cells climbing up vertically to the top of the barriers is shown in Fig. 3a. When cells migrated from a flat surface or grating towards the barriers, the probability of cells climbing up decreased with increasing barrier heights. When the barrier height was increased from 1 to 25 µm, the probability of cells climbing up the barrier decreased from 39.3% to 2.7% and from 56.0% to 10.7% for flat surface and grating, respectively. The higher probability for cells to climb up when they started on a grating than a flat surface suggests that the grating guiding pattern promoted vertical migration of cells. It is well-known that gratings can accelerate cell migration speed and enhance migration directionality^37,40^, making it easier for cells to migrate vertically.

When the barrier height was 1 µm, the vertical climbing probability for cells guided by arrows in channels was lower than cells that started on a flat surface or grating. This low climbing probability may be due to cell interactions in the 10 µm wide channels. When MC3T3 cells migrated in the 10 µm wide channels, they elongated and adhered on both the bottom and sidewalls of the channels. Some of the cells migrated along the channel sidewalls and climbed up to the top of the channel walls. These cells needed to climb down the 11 µm tall channel walls before reaching the 1 µm tall barrier. As a result, the probability of cells climbing over the 1 µm tall barrier for cells guided by arrows in channels was lower than those guided by a flat surface or grating.

The overall vertical climbing probability increased to 33.5% when the barrier height was 5 µm, most similar to the probability of cells climbing up when guided by a grating. The vertical climbing probability decreased when the barrier height was further increased, with cells from the flat surface or grating showing the same trends. The vertical climbing probability of cells on arrows in channels was higher than cells on flat surface or grating when barriers were 10 µm or taller. The vertical climbing probability for cells in channels with arrows was 30.8% and 15.2%, more than 40% higher than cells that started on grating when the barriers were 10 and 25 µm tall, respectively. The enhancement of vertical climbing for cells guided by arrows in channels can be attributed to the improvement of migration directionality due to confinement by the channel sidewalls in the 10 µm wide channels. As cells could only climb up or reverse migration direction when guided by arrows in channels, the vertical climbing probability increased by such guiding pattern.

MC3T3 cells climbing down barriers were also studied in addition to cells climbing up. As shown in Fig. 3b, the ratio of cells climbing up/climbing down is usually larger than 1, meaning that it was easier for cells to climb up than to climb down the barriers. When the barrier was 1 µm tall, the ratio of cells climbing up/climbing down was about 1, indicating that nearly the same number of cells climbed up or down the 1 µm tall barrier. When the barrier height was increased to 5 µm, it was more difficult for cells to climb down to the flat surface compared to when the bottom surface had a grating or arrows in channels pattern. The presence of a 1 µm thick grating or arrows in channels reduced the height difference from the top of the barrier to the bottom surface. It provided more adhesion areas, thus reducing the difficulty for cells to climb down from the top of the 5 µm barrier to the bottom. For 10 µm tall barriers, fewer cells could reach the bottom surface from the top of the barrier. As a result, guiding patterns with grating or arrows in channels showed fewer cells climbing down the barriers than climbing up. When the barrier height was further increased to 25 µm, both climbing up and climbing down became more difficult for the cells. The ratio was reduced to 2.2 and 1.6 for grating and arrows in channels on the bottom surface, respectively. When the bottom was a flat surface, no cells could climb down from the 25 µm tall barrier. On the contrary, a few cells could still climb up from the bottom to the top of the 25 µm tall barrier.

In general, it is more difficult for MC3T3 cells to climb down than climb up the barriers. This may be due to the difficulty for cells to move over the convex angle between the top surface and the barrier sidewall compared to the concave angle between the bottom surface and the sidewall. Several papers have reported how surface curvature regulates cell migration^41–45^. It has been found that cells on a concave surface migrate faster than when they move on a convex surface. Adherent cells prefer to position themselves on a concave surface and avoid migration through convex areas. On convex surfaces, cells fully make contact around the curvature, causing substantial nuclear deformation. On the contrary, cells do not make full contact around the concave curvature. Therefore, MC3T3 cells could climb up the barrier more easily by reaching the barrier sidewall around the concave curvature. Dapi/Hoechst staining of cells on platforms can be used to quantify nuclear deformation on convex or concave surfaces. However, stained cells are static, and they lack the dynamic information needed to distinguish whether the cells are migrating up or down the barriers. Instead, a high-resolution confocal microscope will be used to dynamically track the cell migration around the barriers in the 3D imaging mode in future study to provide more insights in the changes of cell morphology.

### MC3T3 cell migration speed and directionality on engineered patterns

**Figure 4:**
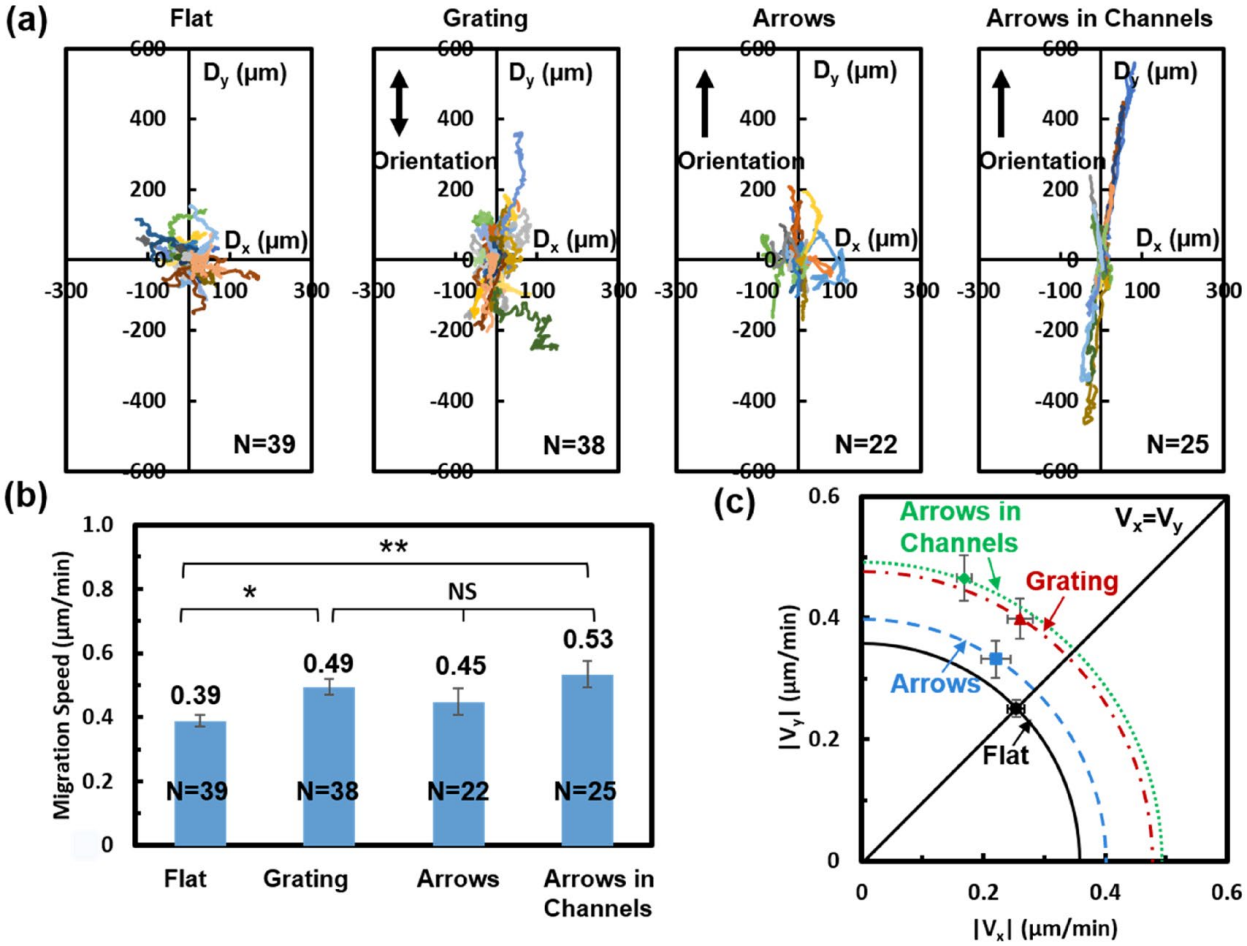
(**a**) Trajectory, (**b**) migration speed, and (**c**) directionality analysis of MC3T3 cells on platforms with flat surface, grating, and arrows in channels. One-way ANOVA and Tukey’s post hoc test were applied to test statistical significance (**p < 0.01, *p < 0.05, and NS – not significant). All the data were obtained over a 16 h time-lapse imaging period.

In order to better understand the cell guiding effects of grating and arrows in channels, cell trajectory, speed, and directionality on these patterns were studied. As shown in Fig. 4a, trajectories of MC3T3 cells on flat surfaces show no directional preference, resulting in random movements. On surfaces with a grating, cells migrated along the y-axis, which corresponds to the grating orientation; this is caused by the guiding effect of gratings on cell migration. Trajectories of cells on the surface with arrows show weak guidance along the arrow direction, while arrows in channels effectively guided the cells along the channels. Cells migrated furthest on the surface with arrows in channels, covering a distance of about 510 µm, while they covered about 370 µm on the flat surface and surface with grating or arrows.

The migration speed of MC3T3 cells on different patterns is shown in Fig. 4b. It was found that cells on arrows in channels migrated fastest, with an average speed of 0.53 μm/min. This is 36% faster than cells on a flat surface, 18% faster than on arrows, and 8% faster than on grating. From the directionality results shown in Fig. 4c, alignment angles for flat surface, grating, arrows, and arrows in channels are 44.6°, 56.1°, 56.5°, and 70.1°, respectively. A larger angle indicates that cells were better guided along the pattern orientation. These results show that cells that migrated on arrows in channels exhibited the best directionality, cells on grating and arrows showed a similar guiding effect, and cells on flat surface migrated randomly. The cell migration direction on a flat surface and grating match previous studies^37,40,46^. The faster and more directional migration of MC3T3 cells on surfaces with grating and arrows in channels helps them climb up taller barriers.

In our previous published work^37^, we focused on the effects of patterned features on osteoblast cell migration on 2D platforms. The controlled patterns included gratings with various depths, bending angles, densities of grating with bends, and discontinuous gratings. In this work, patterned gratings and arrows were used as guiding patterns to promote cell migration over barriers with different heights that were perpendicular to the cell migration direction. These barriers also had various slope angles, and cell migration up or down these barriers was studied. Our previous work focused on enhancing cell migration on 2D patterns, while the main objectives of this work are to investigate 3D cell migration across vertical and sloped barriers.

Focal adhesions (FAs) connect the actin cytoskeleton to the substrate or extracellular matrix, to transmit forces to the substrate. Dynamic FA affects cell adhesion and motility. According to previous works^17^, it was found that with guidance of patterns such as gratings, cells prefer aligning along with gratings, and FA mostly aligns to gratings as well. Cells tend to migrate faster with better directionality with guiding of topographic cues compared with on flat surfaces^34^. When cells migrate towards the barrier with guidance of topographic cues like gratings, cells tend to elongate along the gratings with asymmetric FA on leading edge and trailing tail, which explores and senses the extracellular environment, takes place primarily around the cell front^47^. When cells encounter the vertical barrier, more projection of forward lamellipodia and filopodia can better sense topographic cues from the barrier rather than hindered by the barrier sidewalls immediately. In other words, the more lamellipodia and filopodia sense the barrier, the easier for cells to climb over the barrier. Therefore, directional migration may drive more climb up to the vertical barriers.

### MC3T3 cell migration in vertical direction

**Figure 5:**
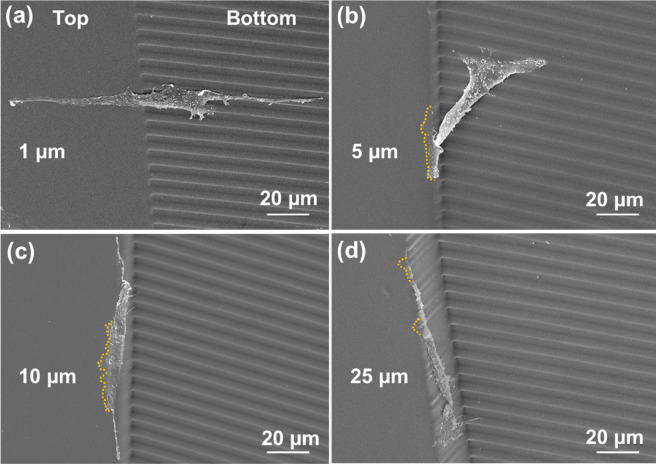
Micrographs of MC3T3 cells on grating pattern with (**a**) 1, (**b**) 5, (**c**) 10, and (**d**) 25 µm tall barriers. Yellow dash lines indicate lamellipodia on top of barriers.

Micrographs of MC3T3 cells on grating next to barriers with different heights were investigated to study how cells migrate vertically. Figure 5a shows a MC3T3 cell elongated along the grating, climbing vertically over the 1 µm tall barrier. When the barrier height was increased to 5 µm, the trailing part of the MC3T3 cell still aligned to the grating while the leading part climbed onto the top of the barrier with a smaller angle long the sidewall compared to the 1 µm tall barrier, as shown in Fig. 5b. When the barrier height was further increased to 10 µm, the MC3T3 cell reached the top of the barrier at a slight angle along the sidewall, as shown in Fig. 5c. With a 25 µm tall barrier, as shown in Fig. 5d, the MC3T3 cell moved along the sidewall of the barrier obliquely to reach the top of the barrier. Cell migration is driven by lamellipodia extensions, which push the cell forwards^48^. When a cell encounters a barrier, the extension of lamellipodia is hampered. Some cells may only contact part of the barrier sidewall when barriers are taller, as shown in Supplementary Fig. S1. As cells need to make surface contact to migrate, insufficient surface contact may make it difficult for cells to reach the top of the barriers^49^. In order to climb up the taller barriers, cells must make full contact with the sidewall, which may cause them to prefer to climb obliquely along the barrier sidewall rather than vertically up the taller barriers.

**Figure 6:**
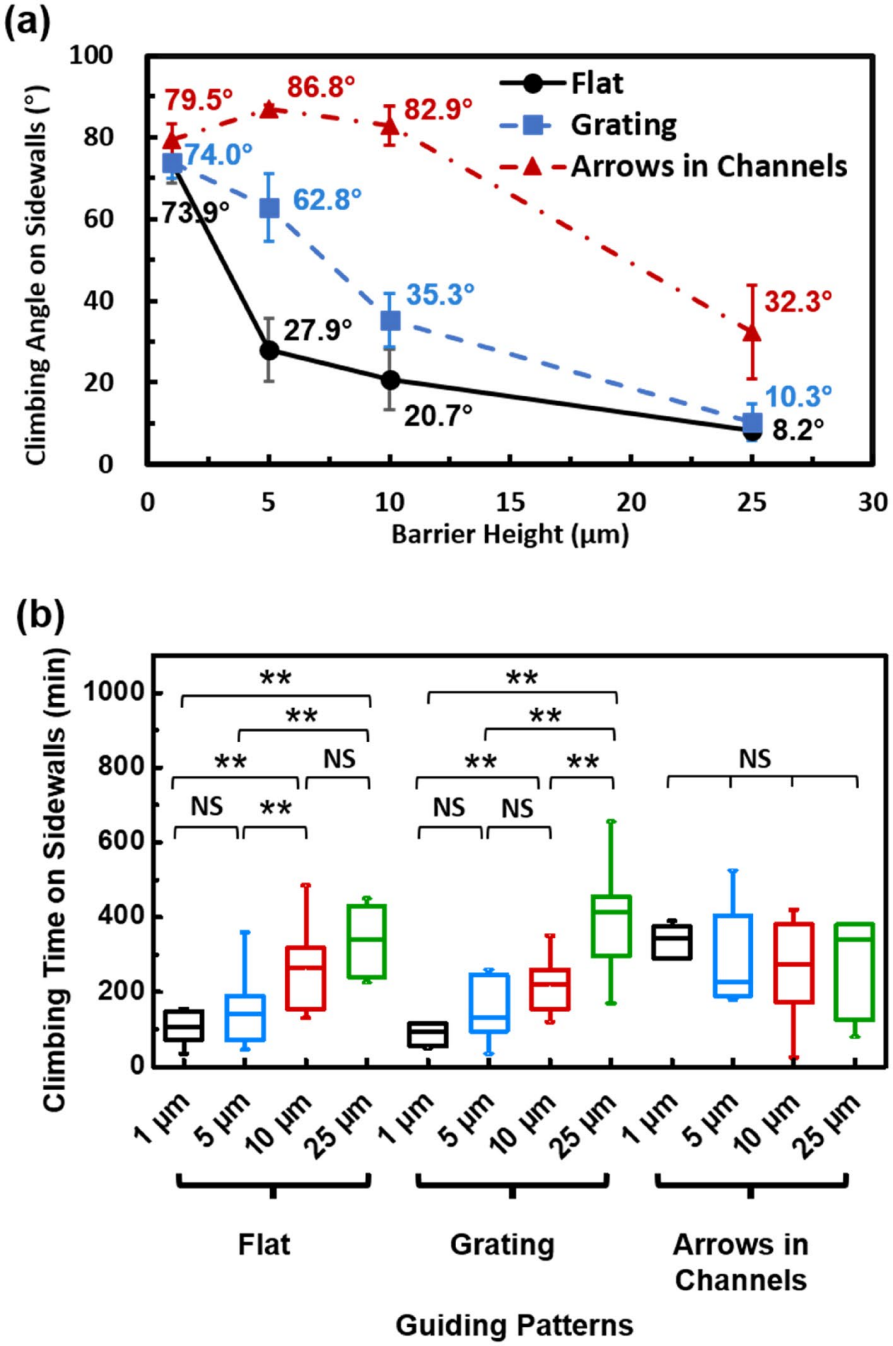
(**a**) Climbing angle of MC3T3 cell trajectory on sidewall of barrier as cells climbed up. (**b**) Time for MC3T3 cells to climb onto top of barriers. One-way ANOVA and Tukey’s post hoc test (**p < 0.01 and NS – not significant). All the data were obtained over a 16 h time-lapse imaging period.

The time-lapse imaging videos as shown in supplementary videos SV2 and SV3 provided dynamic information for how cells climbed up or down the barriers. The climbing angle along the sidewalls of the barriers was calculated, as shown in Fig. 6a. It was found that when the barrier was only 1 μm tall, cells climbed up perpendicular to the barriers. When the barrier height was increased to 5 μm, the climbing angle decreased for cells on a flat surface or grating. When the barrier height was further increased to 25 µm, the climbing angle decreased to 10.3° for cells started on a grating, meaning that the cells climbed along the barrier sidewall. For cells on the surface with arrows in channels, the sideways movement of cells was limited by the channel walls, and the climbing angle was large when the barrier height was 10 μm or less. When the barrier was 25 μm tall, exceeding the 12 μm tall channel walls, some cells moved to the top of the channel walls. Overall, cells from the surface with arrows in channels could climb to the top of the 25 μm tall barrier with a smaller climbing angle of 32.3°, compared to cells climbing over a 10 μm tall barrier with a larger climbing angle of 82.9°.

In addition to the climbing angle, climbing time also indicates the degree of difficulty for cells to climb over a barrier. Climbing time is defined as the time from when a cell touches the bottom of the barrier until the entire cell body reaches the top of the barrier. Figure 6b shows that climbing time increased with taller barriers for cells that started on a flat surface or grating. The results are consistent with the micrographs shown in Fig. 5 and climbing angles shown in Fig. 6a. When the barrier height was low, it took only a short amount of time for cells to climb up to the top of the barriers. For taller barriers, cells contacted the barrier sidewall first and gradually climbed up until its entire body reached the top of the barriers, which resulted in a longer climbing time. For cells that started from a surface with arrows in channels, climbing time varied with barrier height. This may be related to the varying distance between the top of the channel wall and the top of the barrier. Some cells that started from the surface with arrows in channels could travel to the top of the 12 μm tall channel walls. For cells on top of the channel walls, to get over the 1 μm tall barrier, cells needed to climb down 11 μm to get over the 1 μm tall barriers. Likewise, to get over the 10 μm tall barrier, cells needed to climb down 2 μm to the top of the 10 μm tall barriers. Therefore, the probability of getting over the barriers varied due to some cells that climbed on top of the channel walls, which made the distance between the top of the channel walls and the barriers vary with 11, 7, 2, and 13 μm when the barrier height varied from 1, 5, 10, and 25 μm, respectively.

### MC3T3 cell migration modes up and down vertical barrier

**Figure 7:**
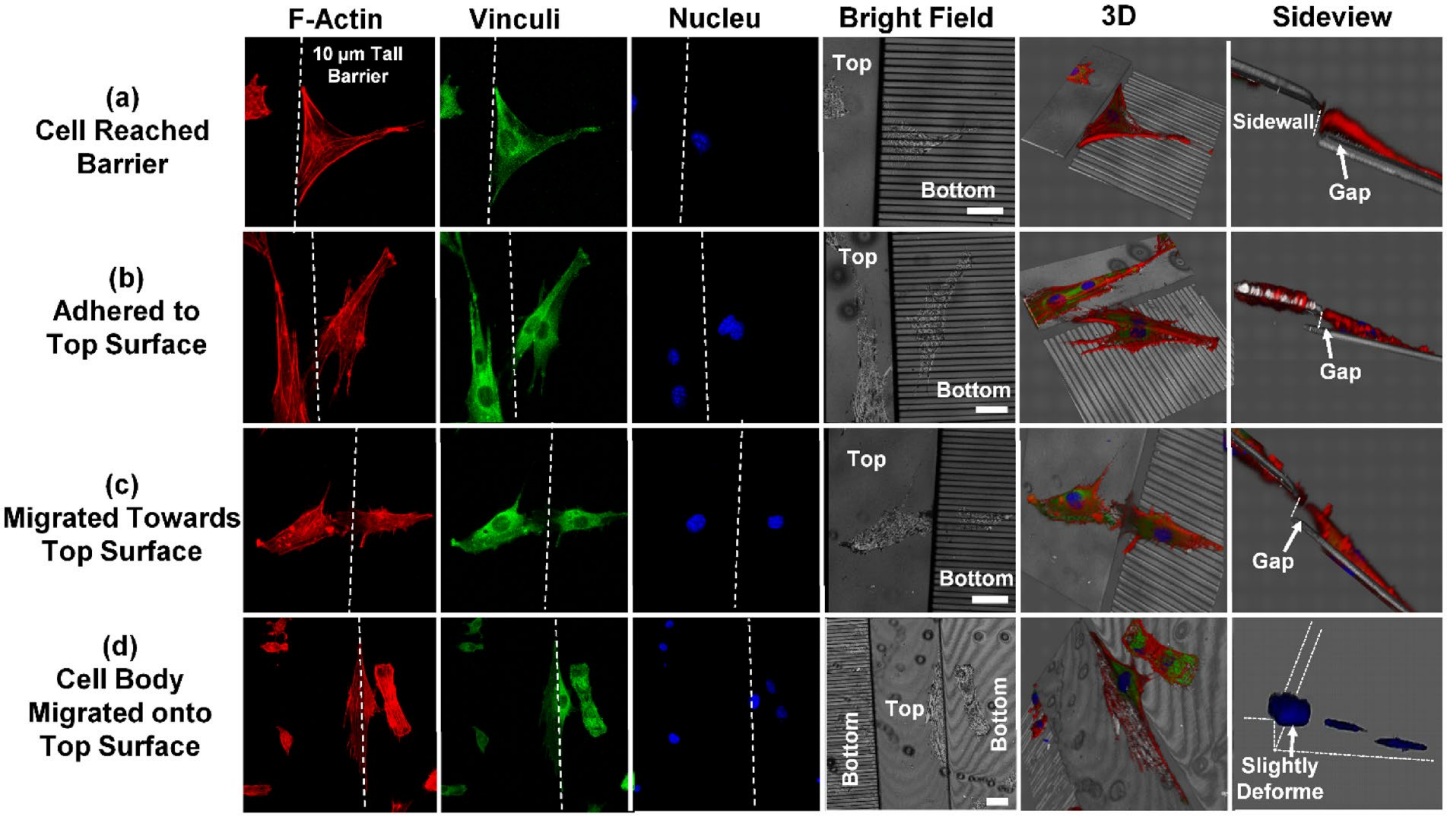
Immunofluorescence micrographs showing MC3T3 cells climbing up 10 µm tall barriers. Red: F-actin; green: vinculin; blue: nucleus; grey: reflected bright field. (**a**) MC3T3 cell on bottom surface reached barrier. (**b**) Cell adhered to top surface and formed elevated morphology. (**c**) Cell aligned and moved towards top surface. (**d**) Cell body and nucleus moved onto top surface. Slight nucleus deformation was observed at barrier edge. All scale bars in the micrographs are 20 µm.

**Figure 8:**
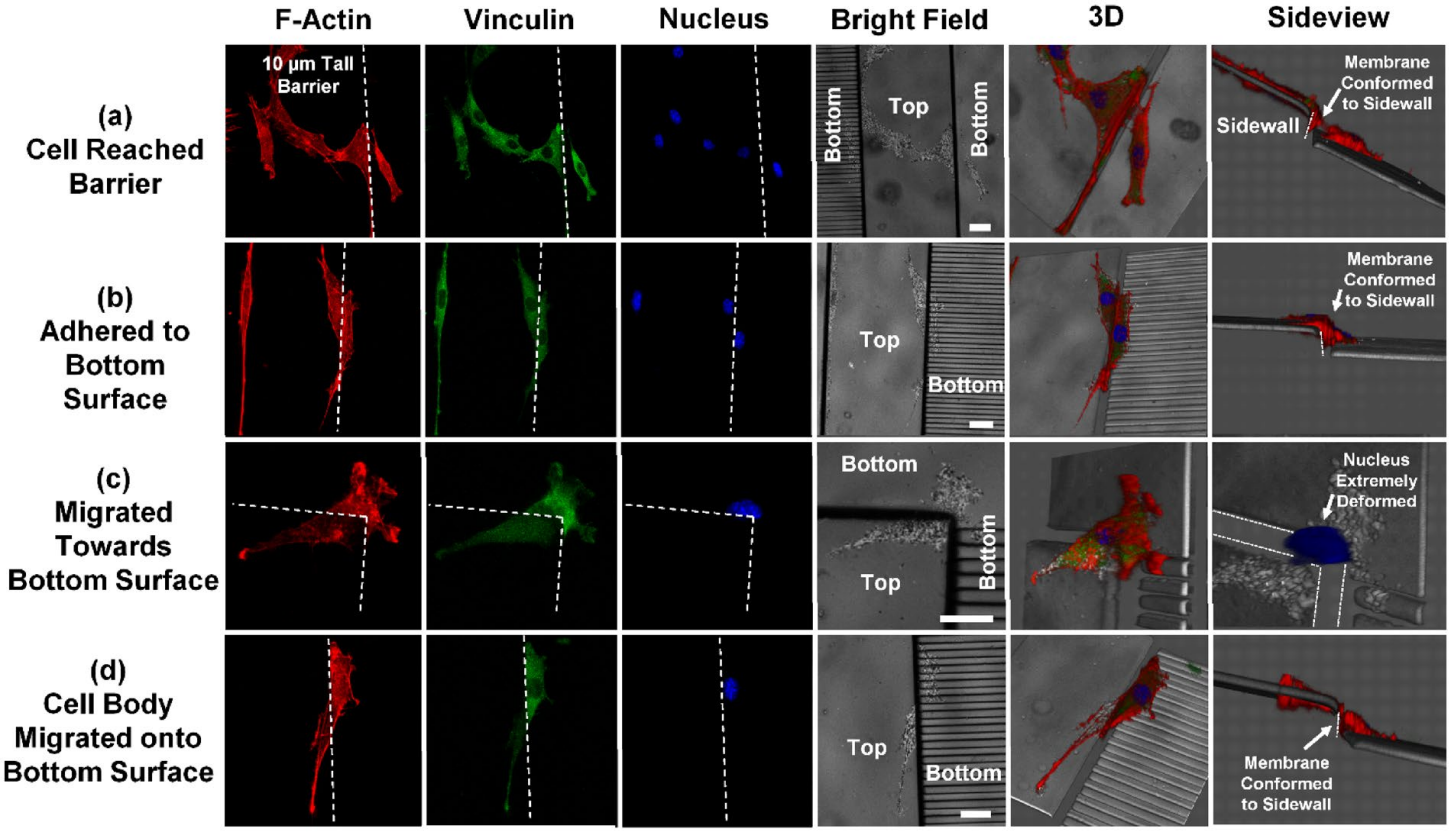
Immunofluorescence micrographs of MC3T3 cells moving down 10 µm tall barriers. Red: F-actin; green: vinculin; blue: nucleus; grey: reflected bright field. (**a**) MC3T3 cell on top surface reached barrier and adhered to sidewall. (**b**) Cell migrated down barrier with cell membrane conformed to sidewall. (**c**) Cells migrated towards bottom surface. Cell nucleus conformed to barrier sidewall and was extremely deformed. (**d**) Cell body moved onto bottom surface. Trailing edge still conformed to sidewall of barrier. All scale bars in the micrographs are 20 µm.

Fluorescence micrographs of MC3T3 cells migrating up and down a 10 µm tall barrier were shown in Figs. 7 and 8, respectively. Projected fluorescence signal, together with the 3D reconstruction of the cell morphology and sideviews of the cells located across the barriers were displayed.

The process of cells moving up or down the 10 µm tall barrier can be shown in 4 steps. When the cell reached the barrier from the bottom surface, the leading edge was aligned to the edge of the barrier as shown in Fig. 7a. The cell then adhered to the top surface. Meanwhile, the adhesions on the sidewall and on part of the bottom surface were released, changing the cell into an elevated morphology as shown in Fig. 7b. The cell did not contact the sidewall conformally, leaving a gap between the sidewall and the cell membrane. The cell then aligned itself and proceed to migrate up the barrier while keeping the elevated morphology as shown in Fig. 7c. In the end, cell nucleus moved up the barrier. Due to the elevated cell morphology, the nucleus was only slightly deformed when crossing the barrier as shown in Fig. 7d.

Cells moving down the barrier followed a similar migration pattern, but the cell morphology during the process was different. Leading edge of the cell aligned to the barrier edge and reached down to the bottom surface as shown in Fig. 8a,b, but the cell membrane conformed to the vertical sidewall. When the nucleus moved down the barrier, it was significantly deformed to fit the shape of the barrier sidewall as shown in Fig. 8c. When the cell body migrated down the barrier to the bottom surface, the trailing edge was still conformed to the sidewall as shown in Fig. 8d.

It was also noted that cells moving up the barrier have clearly defined actin stress fibers during the process. But when cells moved down the barrier, the stress fibers in the leading edge were disrupted. It is suggested that cell migration directionality will be affected due to the deformed cell morphology and disrupted actin stress fibers, making the cells harder to move down a barrier.

### Sloped barrier promoted vertical migration

**Figure 9:**
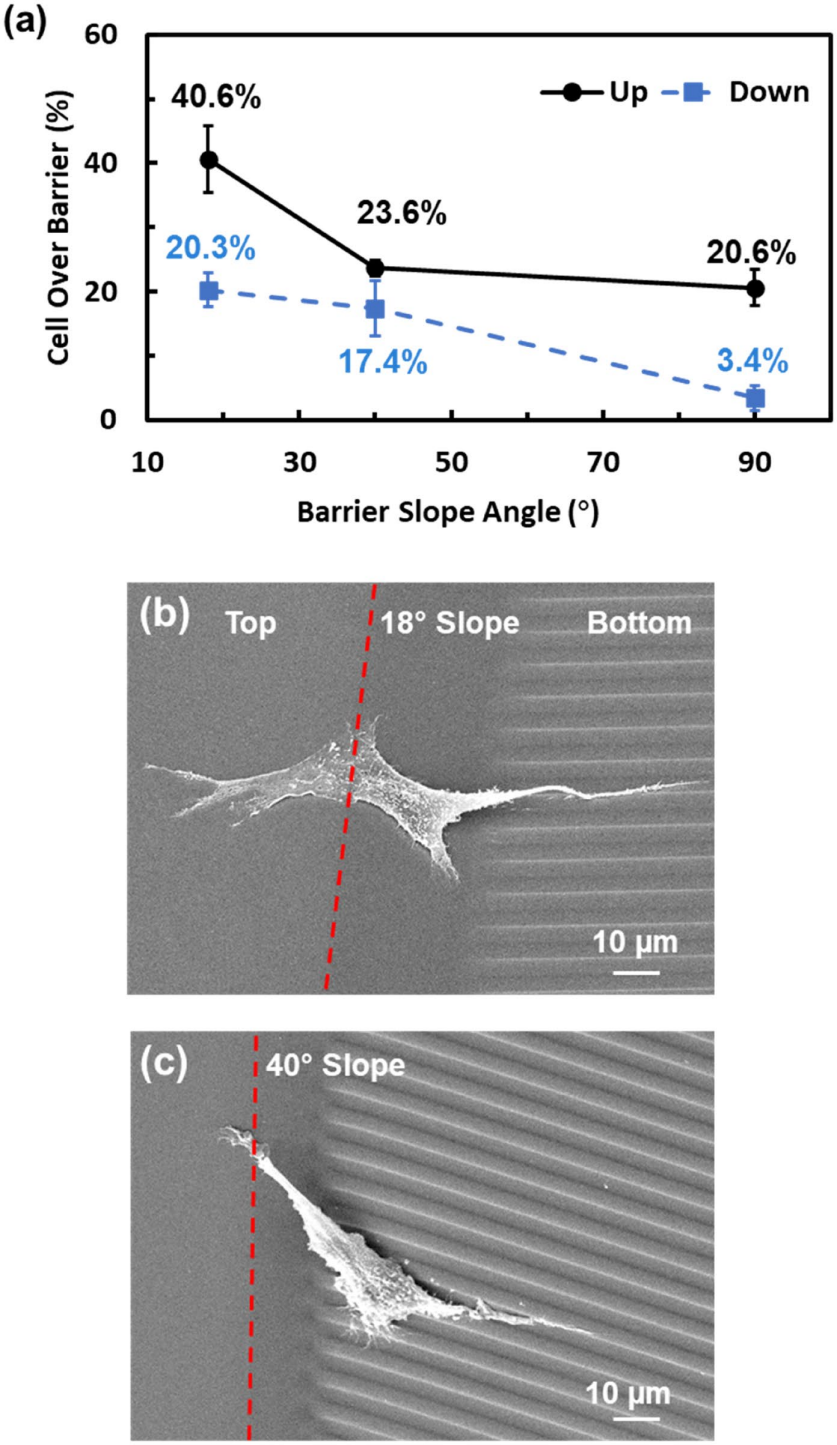
(**a**) Proportion of MC3T3 cells climbed up and down 10 µm tall sloped barriers with slope varying from 18° to 90°. (**b**) Micrographs of MC3T3 cells on 18° and (**c**) 40° slope, 10 µm tall barriers. Red dash lines indicate boundaries of barrier and sloped sidewall. All the data were obtained over a 16 h time-lapse imaging period.

The effects of a sloped barrier on vertical cell migration were studied. As mentioned in Fig. 3a, when the barrier was 10 µm tall, only 20.6% of MC3T3 cells could climb up to the top of the barrier with the help of a grating pattern. Based on this result, sloped barriers with 18° and 40° sidewalls were fabricated next to the 10 µm tall barrier with grating guidance. It was found that 10 µm tall barriers with an 18° sloped sidewall increased the probability of MC3T3 cells climbing up to the top of the barrier from 20.6% for a vertical barrier to 40.6% for a barrier with 18° sloped sidewall, a 97.0% increase as shown in Fig. 9a. Likewise, the probability of MC3T3 cells climbing down from the top of the barrier to the bottom surface increased from 3.4% for the vertical barrier to 20.3% for the 18° sloped barrier, five times the original value. Therefore, barriers with sloped sidewalls can significantly promote the vertical migration of MC3T3 cells.

Micrographs of MC3T3 cells climbing 10 µm tall, 18° and 40° sloped barriers are shown in Fig. 9b,c, respectively. The MC3T3 cell body crossed the 10 µm tall, 18° sloped barrier with the shortest distance. When the barrier had a 40° sloped sidewall, the cell body crossed the barrier sidewall at a small angle. As shown in Fig. 6a, the smaller the climbing angle, the lower the probability for MC3T3 cells to climb up or down when they migrated vertically. As a result, barriers with sloped sidewalls assist the vertical migration of MC3T3 cells.

## Conclusion

In this study, vertical migration of MC3T3 cells was investigated. Grating and arrows in channels guiding patterns were fabricated next to 1, 5, 10, and 25 µm tall barriers to explore the effects of guiding patterns on vertical cell migration. While it is more difficult for cells to climb up taller barriers, the grating pattern increased the probability of cells climbing on top of the barriers. The surface with arrows in channels allowed cells to climb up the barriers more easily when the barrier height was 10 µm or taller. Typically, it was easier for cells to climb up the barriers than to climb down. The cell climbing angle along the barrier sidewall decreased as barrier height increased, whereas climbing time over the barrier increased. The probability of MC3T3 cells climbing up the sloped barriers was 40.6% and 23.6% for 18° and 40° sloped sidewalls, respectively, compared to a probability of 20.6% for a vertical barrier; it is evident that sloped barriers make it easier for cells to migrate vertically. Differences between horizontal and vertical migration are summarized in Table 1. This study provides information for the vertical migration of cells, which is vital for understanding cell migration beyond a 2D horizontal plane.

**Table 1:** Differences between horizontal and vertical migration.

	Vertical migration	Horizontal migration
Number of cells	Less	More
Actin stress fiber	Disrupted when Migrating Down	Normal
Cell nucleus	Deformed during Transition Phase	Normal
Speed	Slower	Faster
Cell shape (aspect ratio)	Larger	Smaller

## Supplementary Information

Supplementary Information 1.

Supplementary Information 2.

Supplementary Information 3.

Supplementary Information 4.
