# Supplementary material for: Engineered barriers regulate osteoblast cell migration in vertical direction
Source: Sci Rep. 2022 Mar 15;12:4459. doi: 10.1038/s41598-022-08262-5 (PMC8924172; doi:10.1038/s41598-022-08262-5)
Supplement: Supplementary file 1 — Supplementary Information 1. [file 41598_2022_8262_MOESM1_ESM.pdf]

Supplementary Information for

"Engineered Barriers Regulate Osteoblast Cell Migration in Vertical Direction"

X. Chen and S. W. Pang*

Department of Electrical Engineering

Centre for Biosystems, Neuroscience, and Nanotechnology

City University of Hong Kong, Kowloon, Hong Kong, China

*Corresponding Author:

S. W. Pang (pang@cityu.edu.hk)

Department of Electrical Engineering

City University of Hong Kong

Kowloon, Hong Kong, China

Phone: +852 3442 9853

Fax: +852 3442 0562

Supplementary Figure

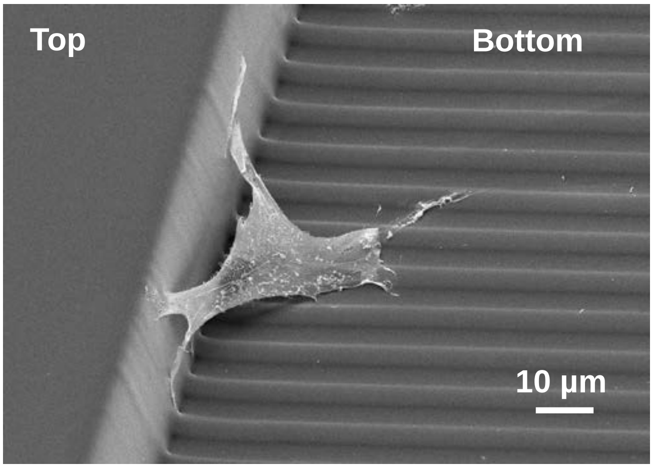

Supplementary Figure S1. MC3T3 cell contacted 25 µm tall vertical barrier.

Supplementary Videos Lengends

Supplementary Video SV1. Cell migration modes when MC3T3 cells encountered 5 µm barriers: (a) cell climbed up to top of barriers, (b) cell moved sideways, and (c) cell reversed migration direction.

Supplementary Video SV2. MC3T3 cells migrating over (a) 10 µm and (b) 25 µm vertical barriers.

Supplementary Video SV3. MC3T3 cells migrating over 10 µm tall barriers with (a) 18° slope and (b) 40° slope.
